# Vestibulospinal tract dysfunction in HTLV-1-asymptomatic infection and in HAM/TSP

**DOI:** 10.1186/1742-4690-11-S1-P26

**Published:** 2014-01-07

**Authors:** Ludimila Labanca, Ana Lúcia B Starling, Silvio Roberto S Pereira, Luiz Cláudio F Romanelli, Anna Bárbara F Carneiro-Proietti, Lucas N Carvalho, Daniele R Fernandes, Denise U Gonçalves

**Affiliations:** 1 HTLV Interdisciplinary Research Group (HTLV-1), Belo Horizonte, Minas Gerais, Brazil; 2 Tropical Medicine Post Graduation Program, Faculty of Medicine, Federal University of Minas Gerais, Belo Horizonte, Minas Gerais, Brazil; 3 Foundation and Center for hematology and hemotherapy of Minas Gerais, Belo Horizonte, Minas Gerais, Brazil

## 

Dizziness is a common complaint in HAM/TSP and can occur due to vestibulospinal tract dysfunction. This tract can be assessed through Vestibular Evoked Myogenic Potential (VEMP). The aim was to correlate the result of VEMP generated by acoustic stimuli and dizziness in individuals with HTLV-1-asymptomatic infection and HAM/TSP. VEMP was recorded from sternocleidomastoid muscle of 60 HTLV-1-negative adults (56 ± 5 years) and 60 individuals infected with HTLV-1, being 30 asymptomatic (56 ± 8 years) and 30 with HAM/TSP (59 ± 8 years). In all groups, 90% of the participants were women. The acoustic stimuli were short tone bursts (1 kHz, 118 dBHL, rise-fall 1 ms, plateau 2 ms), stimulation rate of 5 Hz and the analysis time for each response was 60 ms; 200 responses were averaged for each run. The electromyographic signals were amplified and band-pass filtered between 10 and 1.5 KHz. Of 60 HTLV-1-negative individuals, 14(23%) reported dizziness; VEMP was normal in all. In the HTLV-1-asymptomatic group, 11(37%) complained of dizziness (P=0.22); VEMP was altered in 4(40%) subjects with dizziness and in 1(5%) without dizziness (P=0.05). In the group with HAM/TSP, dizziness was reported by 17(57%) subjects (P=0.002); VEMP was altered in 11(64%) with dizziness and in 5(38%) without dizziness (P=0.15). Damage of vestibulospinal tract seems to occur in the early stages of HAM/TSP. VEMP was previously shown to be a useful test for the follow-up of asymptomatic carriers. Dizziness without an apparent cause in HTLV-1-asymptomatic carriers deserve investigation about a possible spinal cord involvement.

